# *Ex situ* Diet Influences the Bacterial Community Associated with the Skin of Red-Eyed Tree Frogs (*Agalychnis callidryas*)

**DOI:** 10.1371/journal.pone.0085563

**Published:** 2014-01-09

**Authors:** Rachael E. Antwis, Rachel L. Haworth, Daniel J. P. Engelmoer, Victoria Ogilvy, Andrea L. Fidgett, Richard F. Preziosi

**Affiliations:** 1 Faculty of Life Sciences, University of Manchester, Manchester, United Kingdom; 2 Department of Ecological Sciences, Vrije Universiteit Amsterdam, Amsterdam, The Netherlands; 3 FrogLife, Peterborough, United Kingdom; 4 Chester Zoo, Chester, United Kingdom; Graz University of Technology (TU Graz), Austria

## Abstract

Amphibians support symbiotic bacterial communities on their skin that protect against a range of infectious pathogens, including the amphibian chytrid fungus. The conditions under which amphibians are maintained in captivity (e.g. diet, substrate, enrichment) in *ex situ* conservation programmes may affect the composition of the bacterial community. In addition, *ex situ* amphibian populations may support different bacterial communities in comparison to *in situ* populations of the same species. This could have implications for the suitability of populations intended for reintroduction, as well as the success of probiotic bacterial inoculations intended to provide amphibians with a bacterial community that resists invasion by the chytrid fungus. We aimed to investigate the effect of a carotenoid-enriched diet on the culturable bacterial community associated with captive red-eyed tree frogs (*Agalychnis callidryas*) and make comparisons to bacteria isolated from a wild population from the Chiquibul Rainforest in Belize. We successfully showed carotenoid availability influences the overall community composition, species richness and abundance of the bacterial community associated with the skin of captive frogs, with *A. callidryas* fed a carotenoid-enriched diet supporting a greater species richness and abundance of bacteria than those fed a carotenoid-free diet. Our results suggest that availability of carotenoids in the diet of captive frogs is likely to be beneficial for the bacterial community associated with the skin. We also found wild *A. callidryas* hosted more than double the number of different bacterial species than captive frogs with very little commonality between species. This suggests frogs in captivity may support a reduced and diverged bacterial community in comparison to wild populations of the same species, which could have particular relevance for *ex situ* conservation projects.

## Introduction

Symbiotic bacterial communities are commonly found in association with both animals and plants. Often the bacterial community provides some advantage to the host in return for a reciprocal benefit, such as nutrients and a suitable microhabitat in which to live and reproduce [1], [2]. The diversity of bacteria plays an important role in determining the stability and resilience of the community to intrinsic or extrinsic perturbation, such as stress, environmental change, or invasion by pathogens [3]–[6]. For example, Dillon et al. [6] demonstrated a significant inverse relationship between the species richness of the gut of locusts (*Schistocerca gregaria*) and the growth of pathogenic bacteria (*Serratia marcescens*), as well as a significant decrease in the number of infected individuals with an increase in bacterial community diversity.

There is a growing body of research to show the bacterial community associated with the skin of amphibians can influence host susceptibility to a range of infectious diseases (e.g. refs [1], [2], [7]–[12]). Bacteria associated with the skin of amphibians may protect the host from pathogenic infection by; a) increasing competition for space and resources; b) altering the microenvironment of the amphibian skin to prevent colonisation of pathogens, and c) the production of anti-microbials that kill or inhibit the growth of pathogens [1], [11], [13]. For example, Woodhams et al. [9] found that a population of *Rana sierrae* persisting with *Batrochochytrium dendrobatidis* (*Bd*; the amphibian chytrid fungus) in Yosemite National Park (USA) had significantly more individuals with at least one anti-*Bd* bacterial strain associated with their skin than a nearby population of *Rana muscosa* that was naïve to the chytrid fungus and subsequently became extinct when *Bd* spread to the area. Lam et al. [12] compared the same population of *R. sierrae* to another *Bd*-naïve population of *R. muscosa* in the area and found similar proportions of individuals in each population that hosted at least one anti-*Bd* bacterial strain, and this population of *R. muscosa* managed to persist when the chytrid fungus arrived a year later.

The use of anti-*Bd* bacteria as a potential probiotic defense against the chytrid fungus is currently being investigated [14]. It has been shown that potential probiotic bacteria can successfully establish on the skin of amphibians, both directly and via transfer from the environment, with subsequent increases in anti-*Bd* metabolites on the skin, and significantly increased growth and survival on exposure to *Bd* zoospores [15]–[17]. In addition, a range of lactic acid producing bacteria that naturally occur on American bullfrogs (*Lithobates catesbeianus*) have been isolated, and *in vitro* studies suggest inoculating captive populations with these bacteria has the potential to prevent and/or treat bacterial septicemia, either individually or as a multi-strain probiotic [18]–[21]. Therefore the presence of the correct assemblage of bacteria on the skin of amphibians is likely to be important for protecting them from infectious diseases.

Amphibians in the wild have relatively high exposure to bacteria through environmental transmission (e.g. plants, soil, water) and through interactions with both conspecifics (e.g. maternal or paternal transfer, and transfer during mating) and other species [2], [22]–[24]. In contrast, captive amphibians almost certainly interact with fewer individuals, as well as experiencing a less diverse and heterogeneous environment through which to gain bacteria. As a result, captive amphibians are likely to receive lower exposure to a variety of bacteria, and therefore support a simpler cutaneous bacterial community structure in comparison to wild counterparts, making them less resistant to disease on reintroduction to the wild. To the author’s knowledge, there are currently no published studies that compare the native microflora of captive and wild populations of the same species.

Different husbandry conditions in captivity (diet, substrate, enrichment, etc.) are also likely to influence the microbial community. Meyer et al. [25] found that cooler temperatures (10–20°C) led to increased skin sloughing frequency in cane toads (*Rhinella marina*) when compared with warmer temperatures (20–30°C), and that skin sloughing led to a decreased abundance of bacteria on the skin. This indicates that conditions in captivity can indirectly influence the bacterial community, although the direct effects of different husbandry practices are yet to be investigated. For probiotic treatments to be effective, a greater knowledge of the potential influence of captive conditions of the cutaneous bacterial communities is required.

The nutritional status of the host may influence the bacterial community present for two reasons; a) bacteria are likely to utilise the nutrients in the skin mucus of amphibians; and b) there is evidence to suggest the gut of an amphibian acts as reservoir for skin bacteria [2], [26], [27]. Many amphibian species ingest their skin on shedding, thereby also ingesting the cutaneous bacteria, which can then be re-inoculated onto the skin via the cloaca [27]. Diet has been shown to influence the bacterial community associated with the gut of many insect species as well as other host organisms including rainbow trout (*Oncorhynchus mykiss*), Galapagos iguanas (*Amblyrhynchus cristatus, Conolophus subscristatus* and *Conolophus pallidus*), and humans [28]–[32]. The diet of amphibians is also likely to influence the survival of bacteria in the intestinal tract, and therefore influence the community that is re-inoculated onto the skin. This may alter the success of probiotic treatments, as well as potentially making amphibians in captivity and those released back into the wild more vulnerable or resistant to infection from pathogens.

One nutritional aspect of interest to amphibian health is dietary carotenoids [33], [34]. In vertebrates, carotenoids are obtained solely from the diet and act as anti-oxidants in the body as well as precursors for vitamin A [35]. In addition, carotenoids confer red, orange and yellow colouration in many organisms including frogs [36], [37]. In the wild, amphibians have access to a broad range of feeder insects from which to gain carotenoids, whereas captive amphibians are generally fed invertebrates deficient in carotenoids [34]. Gut loading of feeder insects in captivity can be used to increase the availability of carotenoids to amphibians. Red-eyed tree frogs (*Agalychnis callidryas*) fed a carotenoid-enriched diet had significantly greater red colouration in their skin than those fed a carotenoid-free diet [34], indicating that dietary nutrients are deposited in the skin and may therefore have a direct effect on the cutaneous bacterial community.

Here we used culturing methods to compare the cutaneous bacterial community associated with the skin of red-eyed tree frogs (*Agalychnis callidryas*) fed on a carotenoid-enriched diet and a carotenoid-free diet. We also identified bacteria to genus/species level using 16S ribosomal DNA sequencing, and compared these to cultured bacteria collected from a wild population of *A. callidryas* at Las Cuevas Research Station, Chiquibul Rainforest, Belize. We hypothesised that wild *A. callidryas* would support a greater number of bacterial species than captive *A. callidryas*, and that there would be differences in the bacterial species isolated. We also hypothesised that captive *A. callidryas* fed a high carotenoid diet would support a significantly greater bacterial diversity than those fed a carotenoid-free diet.

## Methods

### Ethics Statement

This study was approved by the University of Manchester Ethics Committee and The North of England Zoological Society (Chester Zoo) Ethics Committee, and all methods were non-invasive. Bacteria was collected and exported from the wild population of *Agalychnis callidryas* by permission of Belize Forest Department (Research and Export Permit Number CD/60/3/12) and imported to the UK by permission of DEFRA (Authorisation Number TARP/2012/224).

### Bacterial Sampling from Captive Frogs

Seventeen captive bred *A. callidryas* (F1, originated from pet trade) were fed black crickets (*Gryllus bimaculatus*) gut-loaded with either a high carotenoid diet (5.0 mg/g; n = 10; 5 males, 5 females) or a carotenoid-free diet (0.0 mg/g; n = 7; 4 males, 3 females) (see [34] for details of diets). All frogs were from the same clutch of eggs and were randomly assigned to dietary treatment group at metamorphosis. Thus, observed differences in bacterial communities can be attributed to differences in diet alone. We note that this design is testing for differences between bacterial communities at a given sampling point rather than how bacterial communities change over time in response to the addition of carotenoids to the diet.

Sterile gloves were worn throughout handling and changed for each frog to minimise cross-contamination. The dorsal and ventral regions of the body were swabbed separately to increase the sampling coverage. Frogs were rinsed twice on their dorsal surface using ∼200 ml sterile bottled water to remove any transient bacteria from their skin (as described by [2]). Frogs were then swabbed all over their dorsum to collect cutaneous bacterial communities using sterile Eurotubo® collection swabs (Deltalab, Spain), and the rinsing and swabbing process was then repeated for the ventral surface of each frog. Care was taken to ensure frogs were not harmed during this process, and individuals were monitored for 2 weeks post-swabbing for signs of distress or injury in response to the swabbing, of which none were observed. Swabs were placed into 1.5 ml sterile screw-top tubes containing 1 ml of 1 M NaCl_2_ solution for plating out immediately after swabbing. Tubes containing swabs were vortexed to dissociate bacteria from the swab, then the swab was removed and samples were diluted by pipetting 100 µl into 900 µl of 1 M NaCl_2_. Pilot studies showed plating a dilution of 10^−1^ produced plates with an intermediate amount of growth that was most suitable for assessing the bacterial community (Antwis, unpublished data). Dilutions were individually plated out on low-nutrient R2A agar media (Lab M Ltd., United Kingdom), sealed with parafilm and incubated at 25°C (the same temperature at which captive frogs were maintained). Morphologically distinct bacterial colonies (‘morphotypes’) were counted at days 6 and 12, after which no new colony growth was observed. Representative colonies of each morphotype were then streaked out on R2A agar to obtain a pure culture for DNA sequencing.

### Data Conversion and Statistical Analysis

Bacterial counts for the carotenoid study animals were summed for the two sampling points (days 6 and 12), multiplied by the dilution factor of 10, and the dorsal and ventral surfaces summed for each frog to give a total bacterial community associated with each individual. Data was checked for normality and bacterial abundance was log normalised before analysis. Gender had no statistically significant effect on the bacterial communities associated with frogs, and so males and females were combined for subsequent analyses. Overall bacterial community composition was analysed for differences based on dietary treatment using the Adonis function of the vegan package in RStudio©. Adonis is a permutational multivariate analysis that uses a Bray-Curtis distance matrix based on the abundance of each morphotype to analyse the variation in the overall bacterial community structure. The effect of diet on species richness (the number of different morphotypes isolated from each individual) and total abundance (total number of bacterial colonies isolated from each individual) were analysed using t tests in JMP 10® (data for bacterial abundance were log transformed to achieve a normal distribution).

### Bacterial Sampling from Wild Frogs

Eight *A. callidryas* (four males, four females) were collected from Elegans Pond at Las Cuevas Research Station, Chiquibul Rainforest, Belize (16°43′N, 88°59′W), placed individually in plastic bags, and returned to the research station. Each frog was then rinsed and swabbed as described for the carotenoid diet study animals. Tubes containing swabs were shaken vigorously for 30 seconds, and the contents poured on to R2A agar plates, which were covered in parafilm and bacteria left to grow at ambient temperature for eight days. Samples were not diluted to 10^−1^ (as for the captive frogs) to minimise the number of steps required to grow bacteria in the field. As the purpose of this was to compare the identity of bacteria associated with wild and captive frogs, it is unlikely this had significant effects on the results. Sterile swabs were used to pick representative colonies of each morphotype, which were placed into screw-top tubes containing 1 ml R2A broth media. Tubes were then shipped to the University of Manchester (UK), where the contents of the tubes were poured on to fresh R2A agar plates and incubated at 25°C until bacteria grew. These were then re-streaked to ensure a pure culture was obtained.

### Molecular Methods and Sequencing Analyses

Bacterial species isolated from captive and wild populations of *A. callidryas* were identified using 16S rDNA sequencing with universal primers 27F (5′-GTGCTGCAGAGAGTTTGATCCTGGCTCAG-3′) and 1492R (5′-CACGGATCCTACGGGTACCTTGTTACGACT-3′) (Webster 2003). 16s rDNA fragments were obtained through direct colony PCR amplification (no DNA extraction required) using the GoTaq**®** Colourless Master Mix (Promega Corp., USA) according to the manufacturers instructions. 16S rDNA was amplified using direct colony PCR with the following programme: 95°C for 2 min followed by 30 cycles of 94°C for 30 s, 52°C for 30 s, and 72°C for 90 s, with a final extension step of 5 minutes at 72°C. PCR products were checked for the correct length with gel electrophoresis (∼1500 bp), and then purified with the QIAquick**®** PCR Purification Kit (Qiagen, UK) and sent to Eurofins MWG Operon, Germany for sequencing. A consensus sequence was obtained by combining the forward and reverse sequences in DNA Dynamo Sequence Analysis Software© (BlueTractorSoftware Ltd., UK). Consensus sequences were then blasted against the NCBI database (http://blast.ncbi.nlm.nih.gov/Blast.cgi) to identify each morphotype to genus level (fragment size of ∼1500 bp). Morphotypes with sequence similarity of 99% or greater were considered the same species (reviewed in [38]).

## Results

### Effects of Dietary Carotenoid Availability on the Cutaneous Bacterial Community

A range of 8–13 morphotypes were isolated per individual from frogs fed a carotenoid-enriched diet, and 6–11 morphotypes were isolated per individual from frogs fed a carotenoid-free diet. The Adonis model for diet, gender and the diet × gender interaction showed diet had a significant effect on the overall bacterial community associated with frogs (F_1,13_ = 2.868, p = 0.024), but gender (F_1,13_ = 0.815, p = 0.529) and diet × gender (F_1,13_ = 0.733, p = 0.618) did not. Gender was subsequently removed from the model, leaving a significant effect of diet on the total bacterial community (F_1,15_ = 2.841, p = 0.014).

**Figure 1:**
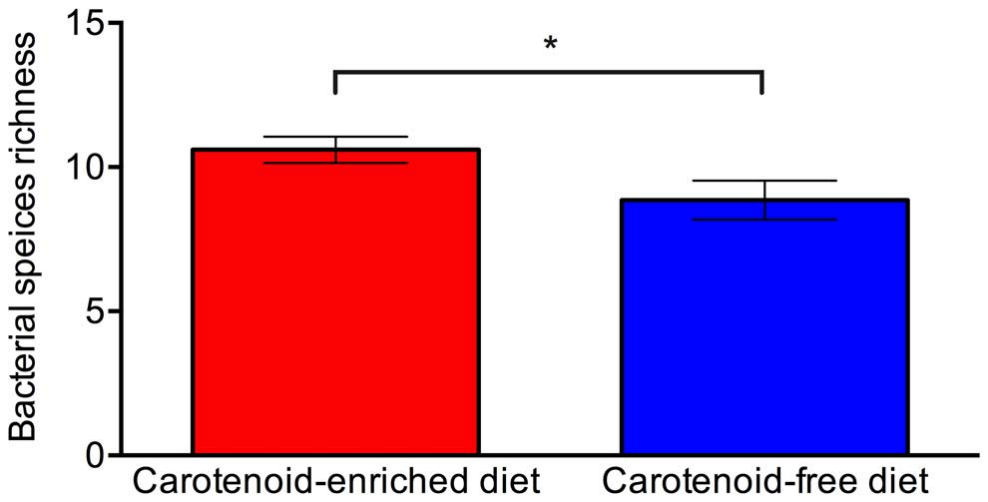
Species richness (number of different bacterial morphotypes isolated from each individual) of the culturable bacterial community associated with the skin of *Agalychnis callidryas* fed a carotenoid-enriched and carotenoid-free diet. Error bars show ±1 S.E.M. An *indicates a significant difference.

**Figure 2:**
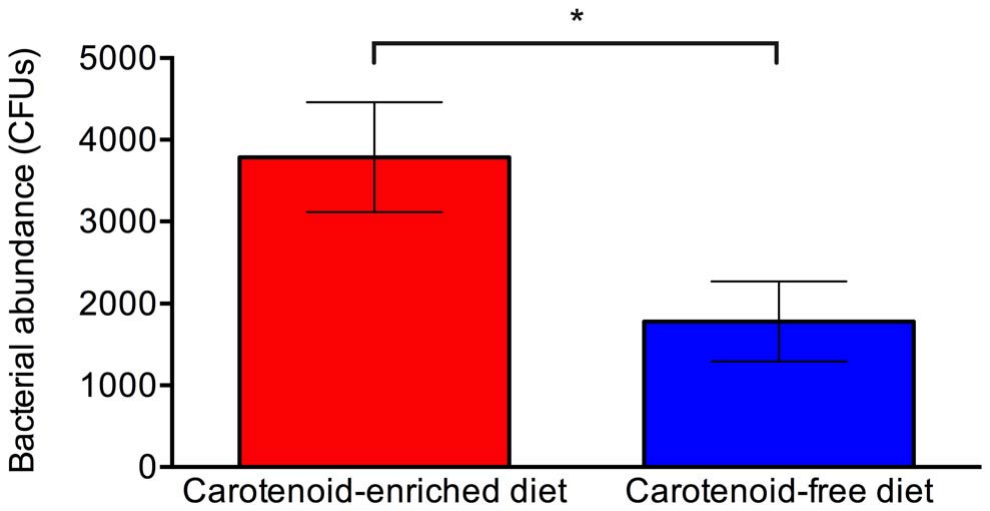
Average bacterial abundance (number of colony forming units (CFUs) isolated from each individual) of the culturable bacterial community associated with the skin of *Agalychnis callidryas* fed on a carotenoid-enriched and carotenoid-free diet. Error bars show ±1 S.E.M. An *indicates a significant difference.

**Figure 3:**
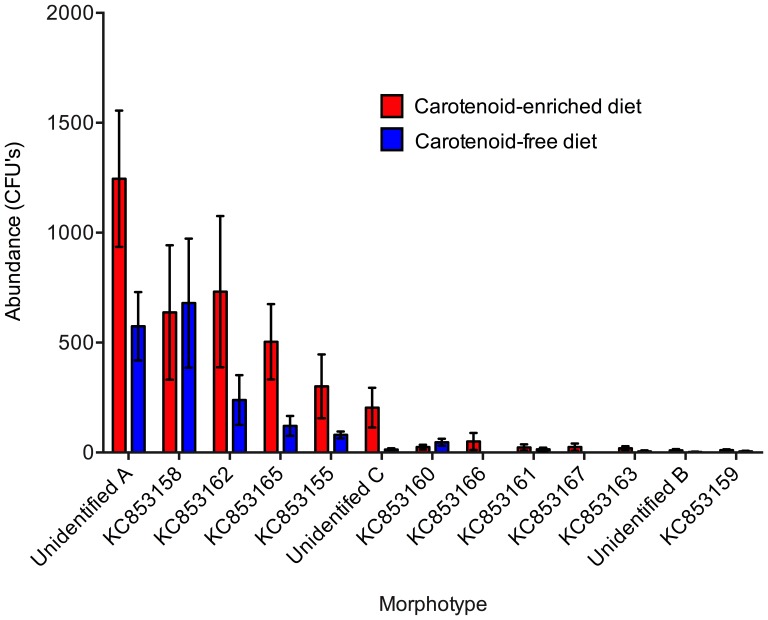
Abundance of each bacterial morphotype (number of colony forming units (CFUs) isolated from each individual) isolated from the skin of *Agalychnis callidryas* fed on a carotenoid-enriched and carotenoid-free diet. Morphotype identities are indicated by GenBank accession number; see Table 1 for details. Error bars show ±1 S.E.M.

**Figure 4:**
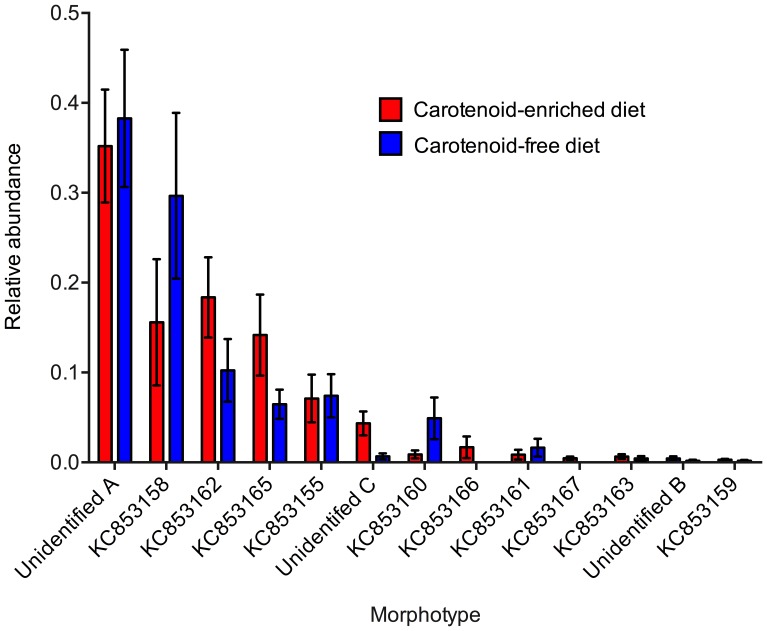
Relative abundance of each bacterial morphotype (number of colony forming units (CFUs) isolated from each individual) isolated from the skin of *Agalychnis callidryas* fed on a carotenoid-enriched and carotenoid-free diet. Morphotype identities are indicated by GenBank accession number; see Table 1 for details. Error bars show ±1 S.E.M.

Gender did not have a significant effect on the bacterial abundance (t_16_ = 0.465, p = 0.324) or species richness (t_16_ = 0.515, p = 0.308) of the bacterial community associated with frogs, and there was no significant interaction between gender and diet, and therefore genders were combined for subsequent analyses. Diet had a significant effect on the bacterial abundance (t_16_ = 2.413, p = 0.015) and species richness (t_16_ = 2.156, p = 0.027) of the bacterial communities associated with frogs, with frogs fed a carotenoid-enriched diet supporting a greater abundance and species richness of bacteria on their skin (Figures 1 and 2). The total abundance (CFU counts) and relative abundance (number of CFUs of each morphotype relative to total number of CFUs isolated from a given individual) of each bacterial morphotype isolated from captive frogs fed the two different diets are shown in Figures 3 and 4. Of the six most abundant morphotypes, frogs fed a carotenoid-enriched diet produced double or more than double the number of colonies for five of those (unidentified A, KC853162, KC853165, KC853155, unidentified C) in comparison to frogs fed a carotenoid-free diet (Figure 3). The abundance of the remaining morphotype was relatively similar between the two groups (KC853158; Figure 3). Figure 4 shows that for these 6 most abundant morphotypes, the relative abundance of each varies according to treatment group. The relative abundance of three morphotypes is greater for frogs fed a carotenoid-enriched diet (KC853162, KC853165 and unidentified C), one morphotype has a greater relative abundance for frogs fed a carotenoid-free diet (KC853158), and two morphotypes have a similar relative abundance between the two groups (unidentified A and KC853155).

### Differences in Bacterial Species between Captive and Wild Populations

Table 1 shows the individual bacterial species isolated from captive and wild *A. callidryas*. Twenty-one morphotypes from 12 families were isolated from the wild populations of *A. callidryas*. Thirteen different bacterial morphotypes (six families) were isolated from frogs fed on a carotenoid-enriched diet, and eleven (five families) were isolated from frogs fed a carotenoid-free diet. Only one bacterial species was isolated from both wild and captive populations (*Stenotrophomonas sp.;* isolated from captive *A. callidryas* fed the carotenoid diet), although three bacterial species isolated from captive frogs could not be identified due to poor sequence data, and colonies were no longer available to resequence. Two dominant families of bacteria were isolated from wild and captive *A. callidryas*; Enterobacteriaceae, and Staphylococcaceae (Table 1). For these two families, more species were isolated from wild frogs than captive (eight vs. two and three vs. two respectively).

**Table 1:** Bacteria isolated from wild *Agalychnis callidryas*, and captive *A. callidryas* fed on a carotenoid-enriched and a carotenoid-free diet.

Family	Species (GenBank accession number;% match to reference strain)	Wild *A. callidryas*	Captive *A. callidryas* fed a carotenoid-enriched diet	Captive *A. callidryas* fed a carotenoid-free diet
*Bacillaceae*	*Bacillus sp.* (KC853209; 99%)	✓		
*Burkholderiaceae*	*Cupriavidus sp.* (KC853214; 98%)	✓		
*Dermabacteraceae*	*Brachybacterium sp.* (KC853155; 99%)		✓	✓
*Deinococcaceae*	*Deinococcus sp.* (KC853204; 98%)	✓		
*Enterobacteriaceae*	*Citrobacter sp.* (KC853165; 99%)		✓	✓
*Enterobacteriaceae*	*Citrobacter sp.* (KC853213; 98%)	✓		
*Enterobacteriaceae*	*Enterobacter sp.* (KC853199; 99%)	✓		
*Enterobacteriaceae*	*Enterobacter sp.* (KC853217; 99%)	✓		
*Enterobacteriaceae*	*Enterobacter sp.* (KC853221; 99%)	✓		
*Enterobacteriaceae*	*Enterobacter sp.* (KC853224; 99%)	✓		
*Enterobacteriaceae*	*Enterobacter sp.* (KC853205; 96%)	✓		
*Enterobacteriaceae*	*Erwinia sp.* (KC853200; 98%)	✓		
*Enterobacteriaceae*	*Klebsiella sp*. (KC853162; 99%)		✓	✓
*Enterobacteriaceae*	*Serratia sp.* (KC853196; 98%)	✓		
*Flavobacteriaceae*	*Chryseobacterium sp*. (KC853218; 97%)	✓		
*Flavobacteriaceae*	*Chryseobacterium sp.* (KC853202; 99%)	✓		
*Flavobacteriaceae*	*Elizabethkingia sp.* (KC853158; 99%)		✓	✓
*Flavobacteriaceae*	*Elizabethkingia sp.* (KC853163; 99%)		✓	✓
*Flavobacteriaceae*	*Flavobacterium sp.* (KC853159; 98%)		✓	✓
*Micrococcaceae*	*Arthrobacter sp.* (KC853208; 99%)	✓		
*Moraxellaceae*	*Acinetobacter sp.* (KC853216; 99%)	✓		
*Pseudomonadaceae*	*Pseudomonas sp.* (KC853219; 99%)	✓		
*Rhizobiaceae*	*Agrobacterium sp.* (KC853210; 98%)	✓		
*Sphingomonadaceae*	*Novosphingobium sp.* (KC853222; 97%)	✓		
*Staphylococcaceae*	*Staphylococcus sp.* (KC853195; 99%)	✓		
*Staphylococcaceae*	*Staphylococcus sp.* (KC853206; 99%)	✓		
*Staphylococcaceae*	*Staphylococcus sp.* (KC853223; 99%)	✓		
*Staphylococcaceae*	*Staphylococcus sp.* (KC853160; 99%)		✓	✓
*Staphylococcaceae*	*Staphylococcus sp.* (KC853161; 99%)		✓	✓
*Staphylococcaceae*	*Staphylococcus sp.* (KC853166; 99%)		✓	
*Xanthomonadaceae*	*Stenotrophomonas sp.* (KC853167/KC853198; 99%)	✓	✓	
*Xanthomonadaceae*	*Stentrophomonas sp.* (KC853220; 99%)	✓		
	*Unidentified A*		✓	✓
	*Unidentified B*		✓	✓
	*Unidentified C*		✓	✓

## Discussion

### Effects of Dietary Carotenoid Availability on the Cutaneous Bacterial Community

Frogs fed a carotenoid-enriched diet had a significantly different bacterial community composition to those fed a carotenoid-free diet. The provision of dietary carotenoids to captive frogs leads to significantly greater number of different bacterial isolates on individual frogs, and a significantly greater abundance of bacteria (Figures 1 and 2). Although the number of different morphotypes isolated from frogs fed a carotenoid-enriched diet were similar to those fed a carotenoid-free diet (13 and 11 respectively), not all frogs supported all these different bacterial morphotypes, and individual frogs fed a carotenoid-enriched diet supported a significantly greater richness of these bacteria compared to individuals fed a carotenoid free diet (Figure 1). Increased species richness and bacterial abundance in a community leads to increased productivity and stability, making the community more resilient to perturbation (e.g. pathogens, stress) and more able to respond to environmental change [1], [3]–[5], [39]. High species richness also increases the likelihood of host individuals harboring at least one bacterial species that confers some protection from infectious pathogens. Therefore frogs fed a high carotenoid diet may possess a bacterial community that is more capable of protecting the host from infectious diseases. Further studies are required to determine the susceptibility to infectious disease of captive amphibians fed different diets.

The increased richness of bacteria on the skin of carotenoid-diet frogs compared to frogs on a carotenoid-free diet suggests the presence of carotenoids could improve the growth and/or survival of bacteria both in the gut and on the skin. *Agalychnis callidryas* ingest their skin on shedding (Antwis, pers. obs.), and greater availability of carotenoids in the diet may also protect the bacteria as they pass through the intestine, thereby allowing them to be re-inoculated on to the skin. For example, some bacteria use carotenoids to protect DNA, proteins and cell membranes from damage by reactive oxygen species that are produced during metabolic processes in the host body [35], [40]. Wiggins et al. [27] isolated the same bacterial species from the gut and skin of *P. cinereus*, and similar genera of bacteria are found in the gut of *R. catesbiana* and *R. pipiens* as are found on the skin of other amphibian species, including *A. callidryas* in this study (e.g. species from the genera *Acinetobacter, Aeromonas, Bacillus, Citrobacter, Enterobacter, Flavobacterium, Klebsiella, Micrococcus, Pseudomonas,* and *Serratia)* [41]–[43]. This suggests bacterial communities associated with the skin and guts of amphibians are of similar origin, and have the potential to influence the composition of one another.

The mucus on the skin of frogs is also likely to be a major source of nutrients for bacteria [2], [26]. The nutritional status of the host may influence the quantity and nutritional composition of the mucus released by mucous glands, as well as the abiotic environment of the skin, which may in turn affect on the bacterial community associated with its skin. In humans, a higher vitamin A content in the blood serum leads to a reduction in sebum content on the skin of humans, and vitamin A and calcium intake has a significant effect on the pH and hydration of the skin, although the impact of this on the bacterial communities has not been investigated [44]. Carotenoids are a precursor to vitamin A, and so the availability of carotenoids in the diet of *A. callidryas* may influence mucus production and/or the microenvironment of the skin. Indeed, short tongue syndrome in amphibians and reptiles is associated with a lack of vitamin A, leading to keratinisation of the glandular epithelium in the tongue and a subsequent reduction in mucus production [18], [33], [39]. It is possible that a similar phenomenon is occurring in the epithelial cells of the skin of *A. callidryas* receiving a carotenoid-free diet, leading to a reduction in mucus availability for subsequent bacterial proliferation.

Two morphotypes isolated from carotenoid-fed frogs were not isolated at all from those fed a carotenoid-free diet (*Staphylococcus sp. KC853166* and *Stenotrophomonas sp. KC853167*; Table 1), although these were found at very low abundance on carotenoid-fed frogs (Figure 3), and so may only play a small role in the bacterial community. For 5 of the 6 most common bacterial morphotypes, frogs fed a carotenoid-enriched diet supported a substantially greater abundance (Figure 3). This may be important for disease prevention in the context of quorum sensing. Quorum sensing is the communication between bacterial cells, via production of signal molecules by the bacteria themselves, which allows communities to initiate physiological processes such as gene transfer, bioluminescence, and anti-microbial peptide production [45]–[47]. For this to occur, a particular density or abundance of bacterial cells is required to obtain a high enough concentration of signal molecules (reviewed in [45]). The role of quorum sensing, or the necessary abundance thresholds of bacteria, has not yet been studied in the context of amphibian defence or probiotic treatment for chytrid disease. Figure 4 shows the relative abundance of each morphotype does not change linearly in bacterial communities of frogs as a result of differences in diet; some morphotypes show an increase in relative abundance with a carotenoid diet, while others decrease or are unaffected. This may also affect community dynamics and responses to pathogenic infection. More work is required to determine the effects of diet, and other *ex situ* environmental conditions, on disease susceptibility of amphibians.

### Differences in Bacterial Species between Wild and Captive Frogs

A much greater number of bacteria were isolated from wild *A. callidryas* than those in captivity. There were taxonomic similarities between the bacterial species isolated from the wild and captive populations at the family and genus level. However, only one bacterial species was isolated from both populations (*Stenotrophomonas sp.*), although three morphotypes from the captive frogs could not be identified due to poor sequence data. Interestingly, the one species in common between captive and wild frogs was only isolated from captive frogs fed a carotenoid-enriched diet, albeit at relatively low abundance. Given that both the community as a whole, as well as some bacterial species in particular, are likely to be involved in protecting the host from invasive pathogens, this may have implications for the ability of captive frogs to protect themselves from pathogens, both in captivity and on reintroduction to the wild, and could have particular relevance for long-term conservation projects involving *ex situ* conservation of amphibians.

Differences in bacterial species between captive and wild *A. callidryas* may be attributed to differences in environment, habitat and diet (as well as many other factors) [1], [13], [51], [52]. Kueneman et al. [50] show that amphibian bacterial assemblage within a species is site specific, with different populations of the same host species supporting different bacterial assemblages. Moreover, 45–76% of operational taxonomic units were shared between the environment (lake water/soil) and the host amphibian [50], indicating the amphibian environment is likely to have a strong effect on the associated bacterial community. This is also likely to be true of the captive environment, particularly in comparison to the wild. The actual species richness is likely to be much higher on both the wild and captive populations of *A. callidryas* as culturing methods only allow a small proportion of the total community to be identified [48]. Greater investigation into the bacteria associated with *A. callidryas* using next-generation sequencing is required to fully understand the true diversity of the community. Individuals from the captive population in this study did not originate from the wild population of *A. callidryas*, and this may explain the lack of shared bacterial species between the two. However, there is growing evidence that shows host species is one of the predominant factors influencing bacterial community assemblage [49], [50], and so we may expect more similarity between bacterial communities isolated from wild and captive populations, irrespective of origin. More work is required to look at how bacterial communities change when host organisms are brought into captivity, and how this may affect their susceptibility to disease.

### Conclusions

This study shows that a diet enriched with carotenoids significantly increases the richness and abundance of bacteria associated with the skin of *A. callidryas*. Given the role of cutaneous bacteria in disease prevention in amphibians, evidence that conditions in captivity have the potential to significantly affect the bacterial community may have implications for the success of probiotic treatments, as well as influence the suitability of amphibians for reintroduction. We also show that captive frogs support a diverged and reduced bacterial community in comparison to wild frogs of the same species. More work is required to determine the effects of other captive conditions on the bacterial communities associated with amphibians, as well as changes in microbiota in response to transfer from the wild to captivity, particularly with regard to the implications for disease resistance and probiotic success.

## References

[1] Belden LK, Harris RN (2007) Infectious diseases in wildlife: The community ecology context. Frontiers in Ecology and the Environment, 5, 533–539 10.1890/060122

[2] Lauer A, Simon MA, Banning JL, André E, Duncan K, et al (2007) Common cutaneous bacteria from the eastern red-backed salamander can inhibit pathogenic fungi. Copeia, 2007, 630–640

[3] Eisenhauer N, Schulz W, Scheu S, Jousset A, Pfrender M (2013) Niche dimensionality links biodiversit and invasibility of microbial communities. Functional Ecology, 27, 282–288 10.1111/j.1365-2435.2012.02060.x

[4] Van Elsas JD, Chiurazzi M, Mallon CA, Elhottova D, Krištufek V, et al (2012) Microbial diversity determines the invasion of soil by a bacterial pathogen. Proceedings of the National Academy of Science, 109, 1159–1164 10.1073/pnas.1109326109 PMC3268289 22232669

[5] Matos A, Kerkhof L, Garland JL (2005) Effects of Microbial Community Diversity on the Survival of Pseudomonas aeruginosa in the Wheat Rhizosphere. Microbial Ecology, 49, 257–264 10.1007/s00248-004-0179-3 15965723

[6] Dillon RJ, Vennard CT, Buckling A, Charnley AK (2005) Diversity of locust gut bacteria protects against pathogen invasion.

[7] Harris RN, James TY, Lauer A, Simon MA, Patel A (2006) Amphibian pathogen *Batrachochytrium dendrobatidis* is inhibited by the cutaneous bacteria of amphibian species. EcoHealth, 3, 53–56 10.1007/s10393-005-0009-1

[8] Lauer A, Simon MA, Banning JL, Lam BA, Harris RN (2008) Diversity of cutaneous bacteria with antifungal activity isolated from female four-toed salamanders. The ISME Journal, 2, 145–57 10.1038/ismej.2007.110 18079731

[9] Woodhams DC, Vredenburg VT, Simon MA, Billheimer D, Shakhtour B, et al (2007) Symbiotic bacteria contribute to innate immune defenses of the threatened mountain yellow-legged frog, *Rana muscosa* . Biological Conservation, 138, 390–398 10.1016/j.biocon.2007.05.004

[10] Pasteris SE, Roig Babot G, Otero MC, Bühler MI, Nader-Macías ME (2009) Beneficial properties of lactic acid bacteria isolated from a *Rana catesbeiana* hatchery. Aquaculture Research, 40, 1605–1615 10.1111/j.1365-2109.2009.02261.x

[11] Becker MH, Harris RN (2010) Cutaneous bacteria of the redback salamander prevent morbidity associated with a lethal disease. PloS One, 5, e10957 10.1371/journal.pone.0010957 20532032 PMC2881031

[12] Lam BA, Walke JB, Vredenburg VT, Harris RN (2010) Proportion of individuals with anti-*Batrachochytrium dendrobatidis* skin bacteria is associated with population persistence in the frog *Rana muscosa* . Biological Conservation, 143, 529–531 10.1016/j.biocon.2009.11.015

[13] Brucker RM, Harris RN, Schwantes CR, Gallaher TN, Flaherty DC, et al (2008) Amphibian chemical defense: Antifungal metabolites of the microsymbiont *Janthinobacterium lividum* on the salamander *Plethodon cinereus* . Journal of Chemical Ecology, 34, 1422–9 10.1007/s10886-008-9555-7 18949519

[14] Bletz MC, Loudon AH, Becker MH, Bell SC, Woodhams DC, et al (2013) Mitigating amphibian chytridiomycosis with bioaugmentation: characteristics of effective probiotics and strategies for their selection and use. Ecology Letters, 16, 807–820 10.1111/ele.12099 23452227

[15] Becker MH, Brucker RM, Schwantes CR, Harris RN, Minbiole KPC (2009) The bacterially produced metabolite violacein is associated with survival of amphibians infected with a lethal fungus. Applied and Environmental Microbiology, 75, 6635–8 10.1128/AEM.01294-09 19717627 PMC2772424

[16] Harris RN, Brucker RM, Walke JB, Becker MH, Schwantes CR, et al (2009) Skin microbes on frogs prevent morbidity and mortality caused by a lethal skin fungus. The ISME Journal, 3, 818–24 10.1038/ismej.2009.27 19322245

[17] Muletz CR, Myers JM, Domangue RJ, Herrick JB, Harris RN (2012) Soil bioaugmentation with amphibian cutaneous bacteria protects amphibian hosts from infection by *Batrachochytrium dendrobatidis* . Biological Conservation, 152, 119–126 10.1016/j.biocon.2012.03.022

[18] Pessier AP (2002) An overview of amphibian skin disease. In: Seminars in avian and exotic pet medicine (Vol. 11, 162–174).

[19] Klaphake E (2009) Bacterial and parasitic diseases of amphibians. The Veterinary Clinics of North America. Exotic Animal Practice, 12, 597–608 10.1016/j.cvex.2009.06.005 19732711

[20] Mendoza GM, Pasteris SE, Ale CE, Otero MC, Bühler MI, et al (2012) Cultivable microbiota of *Lithobates catesbeianus* and advances in the selection of lactic acid bacteria as biological control agents in raniculture. Research in Veterinary Science, 93, 1160–7 10.1016/j.rvsc.2012.05.007 22695175

[21] Pasteris SE, Buhler MI, Nader-Macias ME (2006) Microbiological and histological studies of farmed-bullfrog (*Rana catesbeiana*) tissues displaying red-leg syndrome. Aquaculture, 251, 11–18 10.1016/j.aquaculture.2005.05.007

[22] Walke JB, Harris RN, Reinert LK, Rollins-Smith LA, Woodhams DC (2011) Social immunity in amphibians: Evidence for vertical transmission of innate defenses. Biotropica 43: 396–400 10.1098/rspb.2010.2118

[23] Banning JL, Weddle AL, Wahl GW, Simon MA, Lauer A, et al (2008) Antifungal skin bacteria, embryonic survival, and communal nesting in four-toed salamanders, *Hemidactylium scutatum* . Oecologia, 156, 423–9 10.1007/s00442-008-1002-5 18335251

[24] Soler JJ, Martín Vivaldi M, Peralta-Sánchez JM, Ruiz-Rodríguez M (2009) Antibiotic-producing bacteria as a possible defence of birds against pathogenic microorganisms. Open Ornithology Journal, 2, 29–36 10.1098/8spb

[25] Meyer A, Cramp L, Bernal H, Franklin E (2012) Changes in cutaneous microbial abundance with sloughing: Possible implications for infection and disease in amphibians. Diseases of Aquatic Organisms, 101, 235–242 10.3354/dao02523 23324420

[26] Brizzi R, Delfino G, Pellegrini R (2002) Specialized mucous glands and their possible adaptive role in the males of some species of Rana (Amphibia, Anura). Journal of Morphology, 254, 328–41 10.1002/jmor.10039 12386902

[27] Wiggins PJ, Smith JM, Harris RN, Minbiole KPC (2011) Gut of red-backed salamanders (*Plethodon cinereus*) may serve as a reservoir for an antifungal cutaneous bacterium. Journal of Herpetology, 45, 329–332 10.1670/10-231.1

[28] Hong PY, Wheeler E, Cann IK, Mackie RI (2011) Phylogenetic analysis of the fecal microbial community in herbivorous land and marine iguanas of the Galápagos Islands using 16S rRNA-based pyrosequencing. The ISME Journal, 5, 1461–70 10.1038/ismej.2011.33 21451584 PMC3160690

[29] Colman DR, Toolson EC, Takacs-Vesbach CD (2012) Do diet and taxonomy influence insect gut bacterial communities? Molecular Ecology, 21, 5124–37 10.1111/j.1365-294X.2012.05752.x 22978555

[30] Desai AR, Links MG, Collins SA, Mansfield GS, Drew MD, et al (2012) Effects of plant-based diets on the distal gut microbiome of rainbow trout (*Oncorhynchus mykiss*). Aquaculture, 350–353, 134–142 10.1016/j.aquaculture.2012.04.005

[31] Filocamo A, Nueno-Palop C, Bisignano C, Mandalari G, Narbad A (2012) Effect of garlic powder on the growth of commensal bacteria from the gastrointestinal tract. Phytomedicine: International Journal of Phytotherapy and Phytopharmacology, 19, 707–11 10.1016/j.phymed.2012.02.018 22480662

[32] Martínez I, Lattimer JM, Hubach KL, Case JA, Yang J, et al (2013) Gut microbiome composition is linked to whole grain-induced immunological improvements. The ISME Journal, 7, 269–80 10.1038/ismej.2012.104 23038174 PMC3554403

[33] Ogilvy V, Fidgett AL, Preziosi RF (2011) Differences in carotenoid accumulation among three feeder-cricket species: Implications for carotenoid delivery to captive insectivores. Zoo Biology, 31, 470–8 10.1002/zoo.20416 21866571

[34] Ogilvy V, Preziosi RF, Fidgett AL (2012) A brighter future for frogs? The influence of carotenoids on the health, development and reproductive success of the red-eye tree frog. Animal Conservation, 15, 480–8 10.1111/j.1469-1795.2012.00536.x

[35] Fraser PD, Bramley PM (2004) The biosynthesis and nutritional uses of carotenoids. Progress in Lipid Research, 43, 228–65 10.1016/j.plipres.2003.10.002 15003396

[36] Matsui K, Marunouchi J, Nakamura M (2002) An ultrastructural and carotenoid analysis of the red ventrum of the Japanese newt, *Cynops pyrrhogaster* . Pigment Cell Research, 15, 265–272 12100492 10.1034/j.1600-0749.2002.01085.x

[37] Baruah P, Goswami UC (2012) Characterization of carotenoid pigments in amphibian, rhacophorous bipunctatus. Journal of Research in Biology, 2, 114–118

[38] Janda JM, Abbott SL (2007) 16S rRNA gene sequencing for bacterial identification in the diagnostic laboratory: Pluses, perils, and pitfalls. Journal of Clinical Microbiology, 45, 2761–4 10.1128/JCM.01228-07 17626177 PMC2045242

[39] Miller EA, Green SL, Otto GM, Bouley DM (2001) Suspected hypovitaminosis A in a colony of captive green anoles (*Anolis carolinensis*). Journal of the American Association for Laboratory Animal Science, 40, 18–20 11300682

[40] Tian B, Hua Y (2010) Carotenoid biosynthesis in extremophilic *Deinococcus thermus* bacteria. Trends in Microbiology, 18, 512–20 10.1016/j.tim.2010.07.007 20832321

[41] Carr AH, Amborski RL, Culley DD, Amborski GF (1976) Aerobic bacteria in the intestinal tracts of bullfrogs (*Rana catesbeiana*) maintained at low temperatures. Herpetologica, 32, 239–244

[42] Hird DW, Diesch SL, McKinnell RG, Gorham E, Martin FB, et al (1983) Enterobacteriaceae and *Aeromonas hydrophila* in Minnesota frogs and tadpoles (*Rana pipiens*). Applied and Environmental Microbiology, 46, 1423–1425 6607034 10.1128/aem.46.6.1423-1425.1983 PMC239586

[43] Banas JA, Loesche WJ, Nace GW (1988) Classification and distribution of large intestinal bacteria in nonhibernating and hibernating leopard frogs (*Rana pipiens*). Applied and Environmental Microbiology, 54, 2305–2310 3263838 10.1128/aem.54.9.2305-2310.1988 PMC202854

[44] Boelsma E, van de Vijver LP, Goldbohm RA, Klöpping-Ketelaars IA, Hendriks HF, et al (2003) Human skin condition and its associations with nutrient concentrations in serum and diet. The American Journal of Clinical Nutrition, 77, 348–55 12540393 10.1093/ajcn/77.2.348

[45] Boyen F, Eeckhaut V, Van Immerseel F, Pasmans F, Ducatelle R, et al (2009) Quorum sensing in veterinary pathogens: Mechanisms, clinical importance and future perspectives. Veterinary Microbiology, 135, 187–195 19185433 10.1016/j.vetmic.2008.12.025

[46] Li YH, Tian X (2012) Quorum sensing and bacterial social interactions in biofilms. Sensors, 12, 2519–2538 10.3390/s120302519 22736963 PMC3376616

[47] Salmond GP, Bycroft BW, Stewart GS, Williams P (1995) The bacterial ‘enigma’: cracking the code of cell-cell communication. Molecular Microbiology, 16, 615–624 7476157 10.1111/j.1365-2958.1995.tb02424.x

[48] Kong HH (2011) Skin microbiome: Genomics-based insights into the diversity and role of skin microbes. Trends in Molecular Medicine, 17, 320–8 10.1016/j.molmed.2011.01.013 21376666 PMC3115422

[49] McKenzie VJ, Bowers RM, Fierer N, Knight R, Lauber CL (2012) Co-habiting amphibian species harbor unique skin bacterial communities in wild populations. The ISME Journal, 6, 588–596 21955991 10.1038/ismej.2011.129 PMC3280140

[50] Kueneman JG, Parfrey LW, Woodhams DC, Archer HM, Knight R, et al (2013) The amphibian skin-associated microbiome across species, space and life history stages. Molecular Ecology 10.1111/mec.12510 24171949

[51] Lima AP, Magnusson WE, Williams DG (2000) Differences in diet among frogs and lizards coexisting in subtropical forests of Australia. Journal of Herpetology, 34, 40–46

[52] Mahan RD, Johnson JR (2007) Diet of the gray treefrog (*Hyla versicolor*) in relation to foraging site location. Journal of Herpetology, 41, 16–23

